# Enhanced Pose Estimation for Badminton Players via Improved YOLOv8-Pose with Efficient Local Attention

**DOI:** 10.3390/s25144446

**Published:** 2025-07-17

**Authors:** Yijian Wu, Zewen Chen, Hongxing Zhang, Yulin Yang, Weichao Yi

**Affiliations:** 1 College of Computer Science and Artificial Intelligence, Wenzhou University, Wenzhou 325035, China; 20180188@wzu.edu.cn (Y.W.); 23451350007@stu.wzu.edu.cn (Z.C.); 2 Metaverse and Artificial Intelligence Institute, Wenzhou University, Wenzhou 325000, China; 3220220499@bit.edu.cn (H.Z.); yangyulin1991@gmail.com (Y.Y.)

**Keywords:** YOLOv8-Pose, efficient local attention, sport analytics, badminton scene, human pose estimation

## Abstract

With the rapid development of sports analytics and artificial intelligence, accurate human pose estimation in badminton is becoming increasingly important. However, challenges such as the lack of domain-specific datasets and the complexity of athletes’ movements continue to hinder progress in this area. To address these issues, we propose an enhanced pose estimation framework tailored to badminton players, built upon an improved YOLOv8-Pose architecture. In particular, we introduce an efficient local attention (ELA) mechanism that effectively captures fine-grained spatial dependencies and contextual information, thereby significantly improving the keypoint localization accuracy and overall pose estimation performance. To support this study, we construct a dedicated badminton pose dataset comprising 4000 manually annotated samples, captured using a Microsoft Kinect v2 camera. The raw data undergo careful processing and refinement through a combination of depth-assisted annotation and visual inspection to ensure high-quality ground truth keypoints. Furthermore, we conduct an in-depth comparative analysis of multiple attention modules and their integration strategies within the network, offering generalizable insights to enhance pose estimation models in other sports domains. The experimental results show that the proposed ELA-enhanced YOLOv8-Pose model consistently achieves superior accuracy across multiple evaluation metrics, including the mean squared error (MSE), object keypoint similarity (OKS), and percentage of correct keypoints (PCK), highlighting its effectiveness and potential for broader applications in sports vision tasks.

## 1. Introduction

With the growing popularity of sports and the rapid advancement of artificial intelligence (AI), the integration of AI technologies into athletic training and analysis is becoming increasingly widespread. In professional badminton, the accuracy and efficiency of players’ motions, particularly during actions such as hitting and receiving the shuttlecock, are critical determinants of competitive performance. Traditionally, coaches rely on the manual review of training videos to identify and correct athletes’ technical flaws, a process that is both time-consuming and inherently subjective. In contrast, deep learning-based human pose estimation offers a powerful alternative by enabling the real-time visualization of joint positions during motion. When combined with domain-specific knowledge of human kinematics, such pose data can facilitate the objective and quantitative analysis of athletic performance. Moreover, for spectators, the real-time display of body pose metrics, such as the joint angles between the upper and lower arms or between the wrist and palm, can significantly enhance the overall viewing experience by providing more intuitive insights into the biomechanics of gameplay.

Human pose estimation [1,2] aims to detect the locations of key human body parts and construct structured representations (e.g., skeletal models) from input data such as images and videos [3,4]. Over the past decade, this field has garnered increased attention and has been widely applied in areas such as human–computer interaction, motion analysis [5], augmented reality (AR), and virtual reality (VR). In recent years, a number of highly effective models [6,7,8,9] have been proposed, advancing the state of the art in pose estimation. Among them, several models for human pose evaluation have attracted substantial interest due to their accuracy and practical applicability.

HRNet [10,11] takes high-resolution images as input and consistently maintains high-resolution feature maps throughout the network. By constructing a multi-scale feature pyramid, it effectively preserves both local and global pose information. However, this approach incurs high computational costs, which makes achieving real-time performance a significant challenge. Additionally, HRNet is primarily designed for single-person pose estimation, which limits its applicability in multi-person scenarios. The PersonLab network [12] predicts the short-range and mid-range offsets of each keypoint. Since short-range prediction is less difficult and more accurate, it serves well for heatmap post-processing to obtain more precise keypoint locations. The network employs the Hough transform method [13] to correct keypoint positions. When combined with mid-range offsets, it captures more global skeletal information, thereby producing accurate prediction results. However, due to the structural characteristics of the network, it still faces challenges in handling occlusions. The Hybrid-Pose network [14] adopts stacked hourglass networks to design two convolutional neural network modules: RNet for pose refinement and CNet for pose correction. Before generating the final pose, CNet guides RNet to adjust the joint positions. Yao et al. [15] employ a transformer-based architecture to generate human keypoints aligned with the contextual background of the scene, which provides valuable inspiration for our work. LSDNet [16] represents a lightweight network that uses a Bernoulli distribution to eliminate redundant deep branches without compromising the fusion of multi-scale human feature representations. In addition, it leverages coordinate attention to enhance cross-channel and directional feature representations. By better integrating features across different scales, LSDNet significantly improves the model’s generalization ability. These models achieve remarkable performance in human pose estimation and advance the development of the field. However, they all exhibit certain limitations, particularly in achieving real-time pose estimation under complex and dynamic conditions, such as those encountered in badminton.

With the evolution of deep learning, the You Only Look Once (YOLO) series [17,18,19,20] has emerged as a mainstream solution for object detection tasks, offering an excellent balance between accuracy and computational efficiency. The series has recently progressed to YOLOv8 [21], which introduces notable improvements in accuracy, speed, and model compactness over its predecessors. Building on this foundation, YOLOv8-Pose [1] extends the YOLOv8 architecture to support human pose estimation by integrating keypoint detection modules. This enhancement enables the model to be applied across diverse domains, including industrial inspection and human–computer interaction. In this work, we focus on enhancing the YOLOv8n-Pose model, the lightweight variant of the YOLOv8-Pose series, which is particularly suited for real-time scenarios with limited computational resources. To further improve keypoint localization, we integrate the efficient local attention (ELA) mechanism [22] into the model. ELA addresses the inherent limitations of traditional convolutional operations in capturing long-range dependencies—especially those that are critical for precise keypoint localization—by enhancing the model’s ability to focus on contextually relevant spatial regions. To support this enhancement, we also construct a domain-specific dataset tailored to badminton scenarios, enabling the model to learn from fine-grained motion patterns and poses characteristic of the sport. Experimental results demonstrate that our modified model achieves superior performance in terms of keypoint accuracy and robustness, thereby validating the effectiveness of the proposed approach.

The primary contributions of this paper are as follows.

(1) Construction of a domain-specific dataset: We develop a dedicated dataset for badminton player pose estimation, named the xBHPE dataset, which contains high-quality, annotated samples tailored to the specific movement patterns and challenges of the sport.

(2) Integration of the ELA mechanism: We incorporate the ELA attention module into the YOLOv8-Pose framework, effectively addressing the limitations of traditional convolutional neural networks in modeling long-range dependencies. This integration significantly enhances the accuracy of local keypoint prediction.

(3) Comprehensive evaluation of attention mechanisms: We conduct a comparative analysis of several mainstream attention modules and demonstrate the superior performance of the ELA mechanism. Furthermore, we summarize generalizable optimization strategies regarding the placement of attention mechanisms within feature extraction networks for pose estimation tasks.

## 2. Related Work

Human pose estimation is a fundamental task in computer vision that aims to detect and localize keypoints (e.g., joints) of the human body within images. Despite its importance, it remains a challenging problem due to the complexity of human motion, frequent occlusions, and the limited availability of large-scale, high-quality annotated datasets [23]. Recent advances in 2D pose estimation have achieved remarkable performance on public benchmarks. Existing methods are generally categorized into two main paradigms: bottom-up and top-down approaches [24]. In parallel, numerous pose estimation datasets have been introduced, each tailored to specific application scenarios or motion characteristics. These datasets present unique challenges for model generalization and pose a variety of considerations for performance evaluation.

### 2.1. Human Pose Estimation Datasets

In recent years, deep learning-based human pose estimation methods have made significant advances. Nevertheless, several challenges remain, including limited domain-specific training data, depth ambiguities, and occlusions [2]. The performance of human pose estimation models heavily relies on the quality and diversity of the datasets used for training and evaluation. Currently, three widely adopted benchmark datasets are commonly referenced in the field. (1) COCO Dataset [25]: This dataset contains approximately 200,000 images with over 250,000 annotated human poses. It offers a wide range of pose variations and complex backgrounds, making it a cornerstone for the benchmarking of human pose estimation models in diverse and cluttered scenes. (2) MPII Human Pose Dataset [26]: Comprising more than 20,000 images and about 40,000 annotated human poses, this dataset focuses on everyday human activities and poses. It is frequently used in evaluating models in general and naturalistic settings. (3) Human 3.6M Dataset [27]: This large-scale dataset captures a variety of 3D human activities performed by multiple subjects in a controlled indoor environment. It includes synchronized video and motion capture data, making it a standard for 3D human pose estimation tasks. Despite their broad applicability, these datasets lack sufficient coverage of domain-specific sports scenarios, particularly badminton, which involves fast, dynamic movements and frequent occlusions. To address this limitation, a key contribution of this work is the development of a dedicated dataset tailored specifically to human pose estimation in badminton environments.

### 2.2. Top-Down Pose Estimation Methods

Top-down human pose estimation methods [28] typically adopt a two-stage framework. Initially, a human detector localizes individual human instances within an image. Subsequently, pose estimation is performed on each detected human region. This approach efficiently integrates global contextual information with local appearance features, resulting in enhanced accuracy for keypoint prediction.

However, the inference speed of top-down approaches is directly proportional to the number of individuals in the scene. This limitation restricts their real-time performance in crowded scenarios, as pose estimation must be executed sequentially for each detected instance. Consequently, the computational complexity increases linearly with the crowd density.

For example, Lu et al. [8] propose Regional Multi-Person Pose Estimation (RMPE), a universal framework compatible with various human detectors and single-person pose estimators. RMPE consists of three core components: (1) a symmetric spatial transformer network, which refines inaccurate bounding boxes to obtain high-quality single-person regions; (2) a pose-guided proposal generator, which acts as a data augmentation module during training to enhance diversity; (3) parametric pose non-maximum suppression, which eliminates redundant detections and overlapping pose predictions. While RMPE significantly improves the performance, it remains sensitive to the quality of the initial detection boxes. Errors in detection—such as false positives, missed detections, or inappropriate IoU thresholds—can propagate through the pipeline and negatively affect the pose estimation accuracy.

Subsequently, Chen et al. [29] design the cascaded pyramid network (CPN), which follows a top-down strategy and employs a pyramid structure [30]. The network comprises two cascaded modules: (1) GlobalNet, which handles coarse keypoint detection and performs well for visible joints, and (2) RefineNet, which refines the outputs of GlobalNet, particularly improving predictions of occluded or hard-to-detect keypoints.

Sun et al. [10] propose the high-resolution network (HRNet), which maintains high-resolution representations through parallel multi-scale branches with repeated feature fusion. While HRNet achieves high accuracy in pose estimation, its architectural complexity and sensitivity to hyperparameter tuning present practical challenges.

Xu et al. [31] introduce ViTpose, a transformer-based architecture that reformulates pose estimation by treating the input image as a sequence of patches and leveraging global attention mechanisms. ViTpose achieves high precision in multi-person scenarios, but it exhibits reduced sensitivity to fine-grained local features and incurs high computational costs during training.

Qiu et al. [32] propose DiffusionPose, which formulates 2D human pose estimation as a generative denoising process. By leveraging diffusion models, the method progressively refines noisy initial heatmaps into accurate keypoint predictions. This probabilistic modeling approach offers a novel alternative to traditional deterministic frameworks in pose estimation.

Furthermore, graph convolutional networks (GCNs) [33] are incorporated into human pose estimation by modeling the human skeleton as a graph, where joints represent nodes and bones represent edges. For instance, Qiu et al. [34] employ graph-based representations to depict the human body and use graph reasoning mechanisms to capture spatial dependencies between joints. This structured inference explicitly encodes anatomical relationships and enables the model to leverage topological constraints within the skeleton. Such graph-based frameworks bridge the gap between geometric modeling and deep learning, offering a principled way to incorporate prior knowledge of human body structures.

### 2.3. Bottom-Up Pose Estimation Methods

The bottom-up human keypoint detection algorithm primarily comprises two components: keypoint detection and keypoint grouping (or clustering) [35]. The keypoint detection process is similar to that used for single-person pose estimation; however, it must detect all keypoints of all individuals present in the image, regardless of the category. Once all keypoints are identified, individual human poses are constructed based on these keypoints.

There are generally two common approaches to assembling the human pose skeleton: (1) predicting offset vectors between related keypoints and connecting them accordingly or (2) clustering the detected keypoints such that those belonging to the same person are grouped, while maximizing the separation between keypoints from different individuals. This clustering process ultimately delineates distinct individuals within the scene.

For example, Newell et al. [36] propose a general detection and grouping framework that marks a seminal contribution to multi-person pose estimation using clustering-based methods. Traditionally, multi-person pose estimation involves a two-stage process: detecting all keypoints and then grouping them into individual poses. However, Newell et al. introduce a joint framework that simultaneously handles detection and grouping, based on the observation that these tasks are inherently interdependent. The method uses the hourglass network as the backbone and assigns tags (or embeddings) to keypoints via the heatmap output. Keypoints with similar tags are grouped as belonging to the same individual, while those with different tags are assigned to separate people. This embedding-based clustering enables simultaneous keypoint detection and association.

OpenPose [6], developed by researchers at CMU, introduces a bottom-up approach that has a significant and lasting impact on human pose estimation. OpenPose generates two key outputs from an input image: part confidence maps and part affinity fields (PAFs). Part confidence maps indicate the likelihood of specific keypoints at each pixel location, while PAFs encode the orientation and associations between pairs of keypoints. By combining these outputs, OpenPose applies a bipartite graph-based maximum matching algorithm to assemble detected keypoints into complete human skeletons.

HigherHRNet [37], an extension of HRNet [10] developed by Microsoft, aims to improve the bottom-up pose estimation performance. While HRNet excels in top-down methods due to its parallel multi-resolution design, its application to bottom-up approaches faces challenges, such as variations in person scale and difficulties in localizing keypoints for small individuals. HigherHRNet addresses these issues by first extracting multi-scale feature representations and then upsampling them to generate higher-resolution feature maps. These maps improve the localization of small persons. During training, feature maps at multiple resolutions receive supervision, enhancing the model’s ability to maintain precise keypoint localization across different scales.

The DEKR network [38] introduces an adaptive convolution mechanism that expands the receptive field of each pixel beyond its local neighborhood, allowing the model to focus on keypoint regions. DEKR adopts a multi-branch architecture, where each branch predicts a specific keypoint. This design decouples the feature learning for different keypoints, enabling precise localization. However, this complexity comes at the cost of an increased computational burden.

In contrast, LitePose [39] targets lightweight pose estimation for edge devices. It adopts a single-branch architecture enhanced with large-kernel convolutions, offering a balance between accuracy and latency. This design achieves better performance in resource-constrained environments. Nonetheless, its keypoint regression accuracy still has room for improvement.

## 3. Proposed Method

### 3.1. Architecture Overview

You Only Look Once (YOLO) [18] is a deep learning framework initially developed for real-time object detection. In recent years, it has also shown strong potential in the field of human pose estimation. Compared to traditional multi-stage approaches, YOLO stands out for its high inference speed, low computational overhead, and ability to maintain a balanced trade-off between accuracy and real-time performance, making it particularly well suited for the detection of keypoints in human motion. A key innovation of YOLO lies in its end-to-end architecture, which unifies object detection and keypoint estimation into a single-stage model. Conventional human pose estimation pipelines often involve sequential stages—first detecting human instances, followed by keypoint localization. YOLO, by contrast, eliminates the need for intermediate processing by directly regressing both bounding box coordinates and human keypoints in a single forward pass. This integrated design not only simplifies the workflow but also substantially improves the computational efficiency and latency, thereby facilitating real-time pose analysis in practical scenarios.

**Figure 1:**
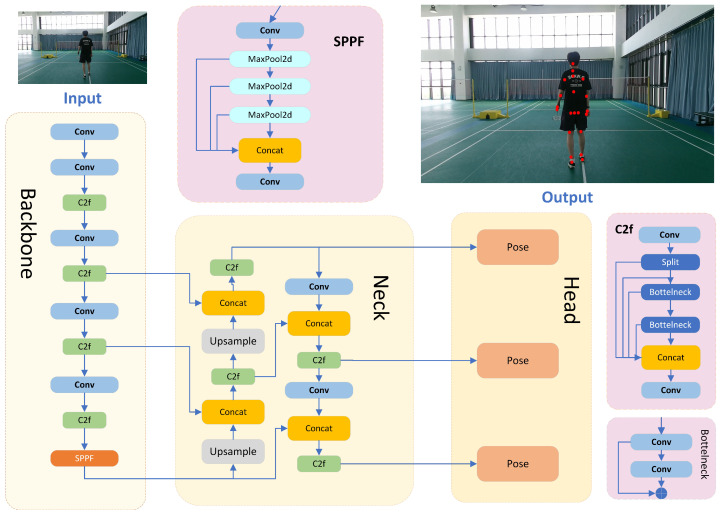
The network architecture of YOLOv8-Pose.

The detailed architecture of YOLOv8-Pose [40] is illustrated in Figure 1. It is mainly composed of three core components: the backbone, neck, and head. The backbone is responsible for extracting multi-scale visual features from the input image, serving as the foundation for downstream tasks. The neck further enhances feature representation by aggregating information across different scales. Finally, the head is specifically designed for keypoint detection, predicting the coordinates and confidence scores of human body joints based on the refined features provided by the previous stages.

**Backbone:** The backbone is primarily responsible for extracting and refining multi-level feature information from input images. Feature extraction plays a crucial role [41], as high-quality feature maps can significantly enhance the prediction performance of neural networks. The backbone consists of three key modules: Conv, C2f, and SPPF. The Conv module is a standard convolutional layer designed to encode images into high-dimensional feature spaces. To reduce the network’s computational cost, a convolutional layer with a 3 × 3 kernel size and a stride of 2 is used as the main feature extraction layer. Additionally, the SiLu non-linear activation function enhances the network’s ability to fit non-linear features. Batch normalization (BN) is applied to standardize features and improve the robustness. The C2f module is a lightweight feature fusion module that allows the network to better utilize multi-scale feature information from different paths or levels, further enhancing the network’s feature representation capabilities. The SPPF module, inspired by the spatial pyramid pooling concept, extracts and integrates spatial features across different feature scales.

**Neck:** The multi-scale neck fusion network is designed to effectively integrate features from different hierarchical levels, enabling the model to capture rich multi-scale contextual information that is essential for precise keypoint localization. This fusion network is composed of three key modules: Concat, C2f, and Upsample. The Concat module performs the channel-wise concatenation of feature maps from different stages, thereby enriching the feature diversity and preserving complementary spatial details. The Upsample module utilizes bilinear interpolation to resample low-resolution feature maps to a higher resolution, facilitating the alignment of spatial dimensions for effective fusion. The C2f module, consistent with its role in the backbone, further strengthens feature representation by introducing additional non-linear transformations and inter-channel interactions, ultimately improving the discriminative power of the detection head. Through the coordinated operation of these modules, the neck network effectively bridges low-level spatial information and high-level semantic features, contributing to more accurate and robust pose estimation performance.

**Head:** The detection head serves as the final output stage of the detection network and consists of three separate subnetworks, each responsible for predictive regression on feature maps at different scales. Unlike previous YOLO versions that employed a coupled head, YOLOv8 adopts a decoupled head architecture, separating the classification and localization tasks. Additionally, the detection head utilizes an anchor-free design, which eliminates errors stemming from improperly designed anchor boxes and avoids the labor-intensive tuning of anchor hyperparameters. This architectural improvement not only reduces the number of model parameters and computational complexity but also enhances the model’s generalization capabilities and robustness across diverse scenarios.

### 3.2. Efficient Local Attention Mechanism

**Figure 2:**
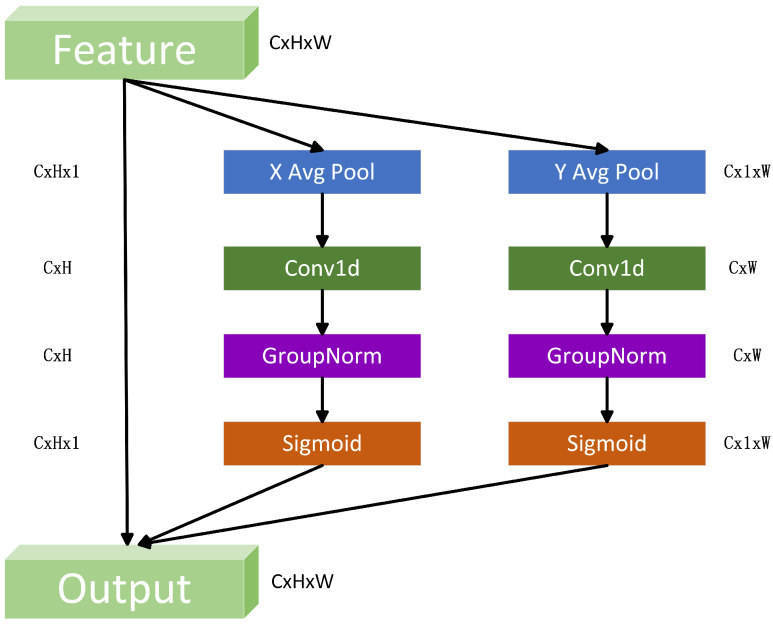
The network architecture of ELA.

Attention mechanisms have garnered substantial acclaim in computer vision for their efficacy in enhancing deep neural network performance. In deep convolutional neural networks (CNNs), these mechanisms are designed to emulate human cognitive behavior, enabling networks to prioritize relevant information while suppressing irrelevant details, thereby augmenting their learning capabilities. Notably, existing attention modules like coordinate attention (CA) [42] and squeeze-and-excitation (SE) [43] exhibit inherent limitations. CA may fail to model long-range spatial dependencies, while SE often compromises channel-wise expressiveness. Such constraints highlight the need for architectural innovations that reconcile the spatial context and channel-wise discriminability in attention-driven frameworks. To address this, the ELA [22] mechanism builds upon the CA module [42] by integrating strip pooling to introduce long-distance spatial dependencies. This design enables the module to accurately localize spatial objects of interest and capture fine-grained positional information about critical regions. The ELA module employs 1D convolutions with kernel sizes of 5 or 7, which offer distinct advantages over 2D convolutions: (1) sequential signal processing, which is better suited for encoding positional interactions in sequential data; (2) parameter efficiency; and (3) fewer learnable parameters compared to 2D convolutions, resulting in lighter-weight architectures. It also has computational economy, reducing the computational complexity while enhancing the positional information flow. The architectural framework of the ELA module is illustrated in Figure 2.

Specifically, Wu et al. [44] observed that the effectiveness of batch normalization (BN) significantly degrades when using small batch sizes. In such cases, the computed batch-wise means and variances may not accurately reflect the overall data distribution, potentially hindering model performance. To address this limitation, the ELA module adopts group normalization (GN) [45] as a regularization strategy. By applying GN (denoted as Gn) to the enhanced positional information, the ELA module effectively captures positional attention across both the horizontal and vertical directions. The final two feature maps, each encoding attention along one spatial axis, are then combined and passed through a sigmoid activation function to generate the output of the ELA module. The detailed calculation procedure of the ELA module is as follows. The input feature has a shape of CxWxH. To apply strip pooling, average pooling is performed on each channel within two spatial ranges: (1) the horizontal direction (H,1) and (2) the vertical direction (1, W). Mathematically, these operations can be expressed as

(1) $$z_{c}^{h}\left(h\right)=\frac{1}{H}\sum_{0\leq i\leq H}x_{c}\left(h,i\right)$$

(2) $$z_{c}^{w}\left(w\right)=\frac{1}{W}\sum_{0\leq j\leq W}x_{c}\left(j,w\right)$$

where $z_{c}^{h}\left(h\right)$ and $z_{c}^{w}\left(w\right)$ denote the horizontal direction and vertical direction results, respectively.

Subsequently, the results undergo processing through 1D convolution, a group normalization (GN) layer, and an activation layer sequentially in two dimensions. This two-dimensional processing pipeline ensures that spatial contextual information is captured and normalized across different feature groups, while the activation layer introduces non-linearity to enhance feature discriminability. This process can be expressed as

(3) $$y^{h}=\sigma \left(G_{n}\left(F_{h}\left(z_{h}\right)\right)\right)$$

(4) $$y^{w}=\sigma \left(G_{n}\left(F_{w}\left(z_{w}\right)\right)\right)$$

where σ represents the activation layer, $G_{n}$ represents the GN layer, $F_{h}$ and $F_{w}$ represent 1D convolution in different directions.

Finally, the output of the ELA module can be expressed as follows:

(5) $$Y=x_{c}\times y^{h}\times y^{w}$$

where *Y* represents the final feature result. This formulation enables the ELA module to selectively emphasize informative regions while suppressing irrelevant background noise, thereby enhancing the spatial sensitivity of the pose estimation model. The integration of GN ensures stable training across varying batch sizes, making the ELA module a lightweight yet effective addition to the YOLOv8-Pose framework.

### 3.3. Loss Function

The YOLOv8-Pose network seamlessly integrates both object detection and keypoint estimation within a unified architecture. Its loss function is composed of the following four components, each targeting a specific aspect of the prediction task.

**Bounding Box Regression Loss.** The bounding box regression loss is used to calculate the difference between the predicted bounding box and the true bounding box. YOLOv8-Pose typically uses a CIoU loss [46,47], combining similarities in the IoU, center point distance, and aspect ratio.

(6) $$L_{bbox}=1-CIoU\left(b,\hat{b}\right)$$

where *b* is the predicted boundary box, and $\hat{b}$ is the real boundary box.

**Objectness Loss.** The target confidence loss is used to measure whether the model correctly predicts the existence of the target. A common loss function is the binary cross-entropy loss.

(7) $$L_{obj}=-\left[y_{obj}\log\left({\hat{p}}_{obj}\right)+\left(1-y_{obj}\right)\log\left(1-{\hat{p}}_{obj}\right)\right]$$

where $y_{obj}$ is the real target existence mark, and ${\hat{p}}_{obj}$ is the target confidence predicted by the model.

**Classification Loss.** The classification loss is used to calculate the error of the model in the prediction of the target class. YOLOv8 also commonly uses binary cross-entropy losses or multiclass cross-entropy losses.

(8) $$L_{cls}=-\sum_{c\in classes}y_{c}\log\left({\hat{p}}_{c}\right)$$

where $y_{c}$ is the unique thermal coding of the real class, and ${\hat{p}}_{c}$ is the class probability predicted by the model.

**Keypoint Regression Loss.** The keypoint regression loss is used to measure the difference between the model’s predicted keypoint location and the true keypoint. Common loss functions are the L2 loss or L1 loss. In our work, we use the L2 loss, as shown in Equation (9):

(9) $$L_{keypoint}=\sum_{i=1}^{N}\left|\right|k_{i}-{\hat{k}}_{i}{\left|\right|}_{2}^{2}$$

where $k_{i}$ is the coordinate of the *i*th true keypoint, and ${\hat{k}}_{i}$ is the coordinate of the *i*th keypoint.

**Total Loss.** The total loss function of YOLOv8-Pose is the weighted sum of the above loss functions. Each loss function is assigned a weight to balance their contributions to the final loss:

(10) $$L_{total}=\lambda_{bbox}L_{bbox}+\lambda_{obj}L_{obj}+\lambda_{cls}L_{cls}+\lambda_{keypoint}L_{keypoint}$$

where $\lambda_{bbox}$, $\lambda_{obj}$, $\lambda_{cls}$, and $\lambda_{keypoint}$ denote the balancing weights for the bounding box, objectness, classification, and keypoint losses, respectively.

The YOLOv8-Pose loss function is designed to comprehensively address both target detection and keypoint detection requirements. By combining and weighting the losses from bounding box regression, target confidence, classification, and keypoint regression, the model is optimized to effectively perform both object detection and pose estimation tasks. These loss components interact during training, allowing the model to learn precise bounding boxes, accurate classifications, and correct keypoint locations.

## 4. Experiments

### 4.1. Experimental Setting

This study uses an Intel(R) Core(TM) i7-9700 CPU @ 3.00 GHz, an RTX A6000 GPU with 48 GB of memory, and the Ubuntu 18.04 64-bit operating system. The deep learning framework is PyTorch 2.1.1, and the unified computing architecture is CUDA 12.1. During training, the Adam optimizer is employed with beta parameters set to 0.9 and 0.999. The batch size is set to 16, the input image size is 1920 × 1080, and the initial learning rate is 0.0001. A linear learning rate decay strategy is adopted, gradually reducing the learning rate to 0 over the course of the training epochs. The model employs the Task-Aligned Assigner [48] during the training process.

### 4.2. Methodology for Dataset Creation

We use Microsoft’s Kinect camera to construct the dataset. The Kinect is a 3D motion-sensing device that offers features such as real-time motion capture, impact recognition, microphone input, speech recognition, and support for interactive applications. The specific parameters of the Kinect camera are presented in Table 1.

The Kinect camera employs an infrared (IR) projector that actively emits near-infrared light. When this light strikes a rough surface or passes through diffusive materials such as ground glass, it becomes distorted, forming random patterns of reflection known as speckles. These speckles are captured by the depth sensor, which analyzes the infrared patterns to generate a detailed depth map of the scene.

**Figure 3:**
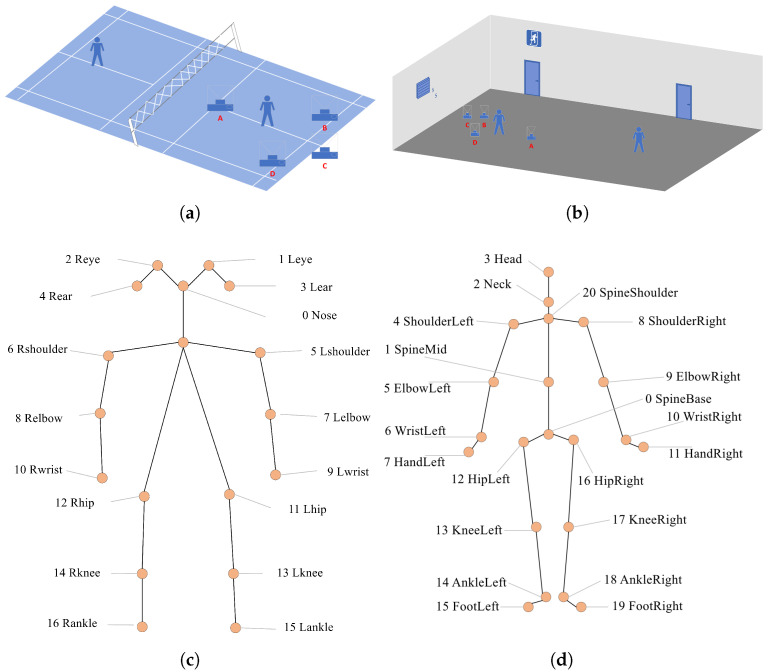
The location and keypoints of the person in the shooting dataset. (**a**) Professional badminton court shooting schematic. (**b**) Non-professional badminton court shooting schematic. (**c**) Seventeen-keypoint skeleton model. (**d**) Twenty-one-keypoint skeleton model.

In our system, we leverage Python to access the Kinect v2 APIs on the Windows platform to retrieve both high-resolution color images and skeletal joint data. The color images are recorded at a resolution of 1920 × 1080 pixels. To maximize the diversity and representativeness of the dataset, we collect data in both professional indoor badminton courts (Figure 3a) and standard indoor environments (Figure 3b), simulating both professional and amateur badminton scenarios. As depicted in Figure 3, the Kinect camera is placed at four distinct angles relative to the athlete: directly in front, directly behind, 45° to the left, and 45° to the right. This multi-view setup ensures the comprehensive capture of body movement from different perspectives. While existing pose estimation datasets typically adopt a 17-keypoint skeletal model (Figure 3c), which includes facial landmarks, such details are generally less relevant in sports applications. In response, we propose an enhanced 21-keypoint skeleton model that places greater emphasis on full-body poses, particularly in capturing dynamic motion, as shown in Figure 3d.

**Figure 4:**
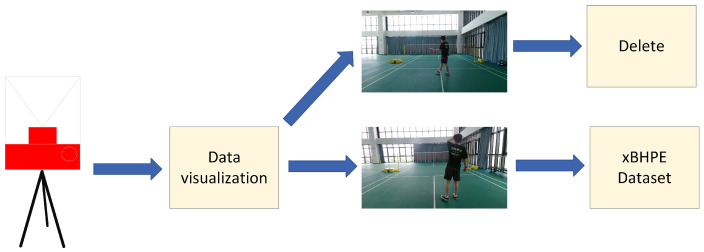
Schematic diagram of the dataset collection process.

Figure 4 illustrates the data generation pipeline of the xBHPE dataset. Raw data are initially captured using a Microsoft Kinect v2 camera, which provides both depth information and skeletal joint data. For each frame, skeletal keypoints are visualized to facilitate manual inspection. Based on these visualizations, a manual screening process is conducted to determine whether each data sample should be retained or discarded. For instance, the sample image shown in Figure 4 is excluded because several keypoints, particularly those on the arms and legs, are either missing or inaccurately positioned due to occlusion or overlapping body parts, resulting in unreliable pose information. This manual verification step plays a crucial role in ensuring the accuracy and quality of the pose annotations by filtering out samples affected by occlusion, incorrect detections, or ambiguous body configurations.

**Figure 5:**
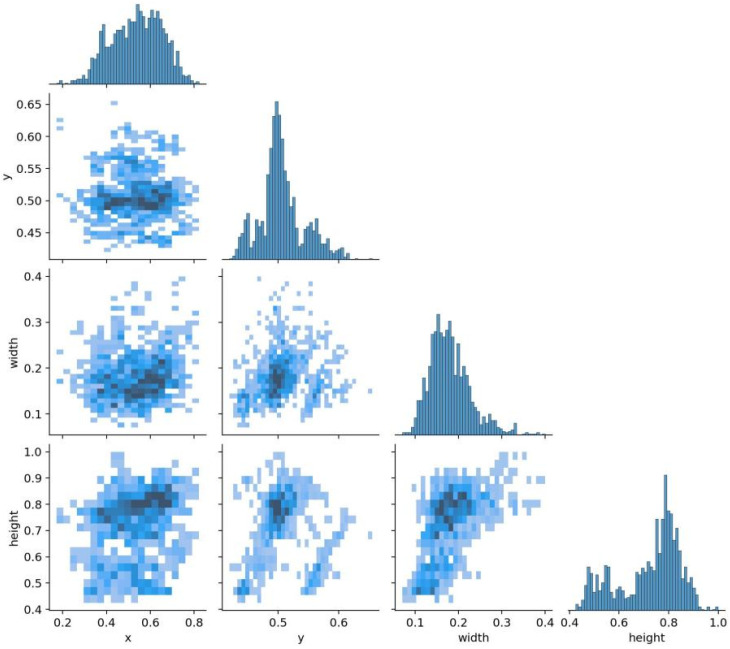
Analysis diagram of the collected dataset.

Figure 5 presents the distribution of bounding box annotations in our constructed dataset. Each subplot visualizes the relationships among four key variables commonly used in object detection tasks: the bounding box center coordinates (x, y), width, and height. As illustrated, the distributions of the x and y coordinates are heavily concentrated near the image center, indicating that most annotated subjects appear centrally within the frame. This spatial bias aligns with typical patterns observed in human-centric datasets, particularly in sports or surveillance scenarios, where subjects are usually captured near the center of the scene. The distribution of the bounding box widths is relatively narrow, while the heights are primarily concentrated within the range of 0.5 to 0.9 (normalized values), suggesting that most annotated objects exhibit a vertically elongated shape. This conforms to the natural aspect ratio of the human body, especially in upright or dynamic motion poses, and is well suited to pose estimation tasks. The scatter plot of the bounding box width versus height reveals a clear positive correlation, indicating that taller individuals tend to have proportionally wider bounding boxes, which reflects standard human anatomical proportions. In contrast, the relationships between x and the width, as well as y and the height, appear more dispersed, implying that the object size is not strongly dependent on the spatial location within the frame. This variability in pose, position, and scale contributes to the diversity of the dataset, thereby enhancing its potential to support the development of more robust and generalizable pose estimation models.

In summary, this dataset exhibits the following characteristics. (1) Centralized Target Distribution: Most annotated targets are located near the center of the image, which is consistent with common practices in human detection and ensures alignment with standard detection scenarios. (2) Reasonable Scale and Aspect Ratios: The bounding boxes exhibit aspect ratios that closely match the proportions of the human body and other vertically oriented objects. This characteristic supports the dataset’s suitability for human pose estimation tasks. (3) Structural Diversity: Although the spatial distribution of targets is relatively centralized, the variability in the bounding box width-to-height ratios introduces diversity in the target shapes. This enhances the model’s ability to generalize across different poses and body types.

### 4.3. Fine-Tuned YOLOv8-Pose with ELA

**Figure 6:**
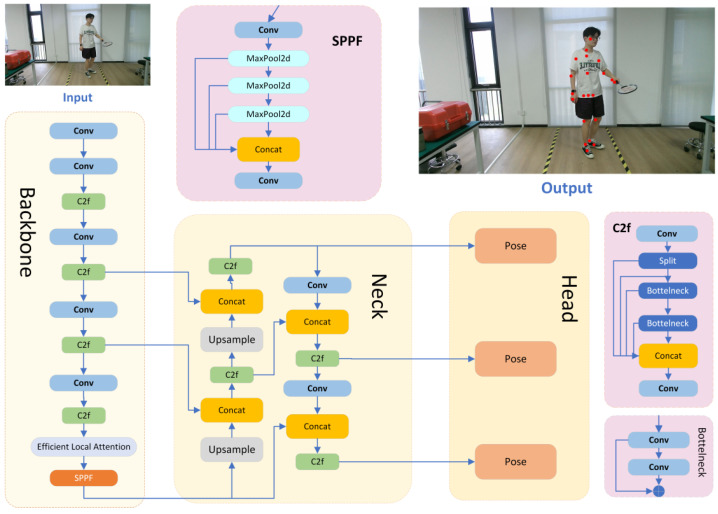
The network architecture of YOLOv8-Pose with ELA.

The primary function of the ELA mechanism is to improve feature extraction. In the YOLOv8-Pose network, the backbone component is tasked with extracting hierarchical features from the input images. Its final layer, the spatial pyramid pooling fast (SPPF) module, is specifically designed to accommodate inputs of varying resolutions while producing feature maps of a fixed size. To fully leverage the capabilities of ELA, we integrate the ELA module immediately before the SPPF layer. This strategic placement allows the ELA module to refine the features generated by the preceding convolutional and C2f modules, thereby enhancing the quality of the extracted representations. The final architecture of the fine-tuned network, incorporating the ELA module, is illustrated in Figure 6.

Specifically, the ELA module is designed to strengthen the model’s ability to capture fine-grained local features while maintaining efficiency. Traditional convolutional operations often struggle to balance local detail preservation with contextual awareness, especially in tasks like human pose estimation, where precise keypoint localization, such as the joints, wrists, and elbows, is critical. The ELA module addresses this challenge through a lightweight 1D grouped convolution structure, which enhances channel-wise feature representation while preserving spatial details. Additionally, by integrating group normalization (GN), the module ensures stable feature distribution across different mini-batches, making it more robust to varied input scales and poses. Placing ELA just before the SPPF layer allows it to refine intermediate features after the early-stage convolution and C2f blocks, ensuring that the most informative local patterns are emphasized before multi-scale aggregation takes place. This strategic positioning not only improves the network’s spatial sensitivity but also enables better generalization in complex pose scenarios involving occlusion, deformation, or low visibility.

To optimize the model architecture, we conduct a series of experiments exploring different parameter combinations, with the results summarized in Table 2. For instance, the model variant labeled ELA-7-256-16 indicates the use of a kernel size of 7 for the 1D convolution, 256 convolutional groups, and 16 groups in the group normalization (GN) layer. Through extensive experimentation, we identify the most effective parameter settings tailored to the YOLOv8-Pose backbone, which allow the model to achieve optimal performance. These findings highlight the critical role of careful module parameterization in maximizing the pose estimation accuracy.

**Figure 7:**
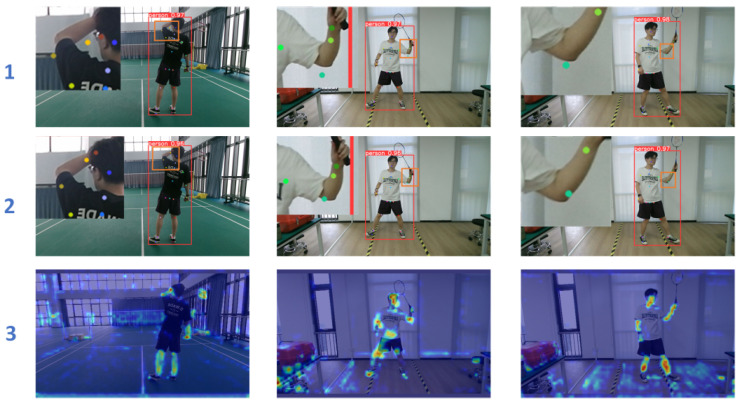
Comparison of visualization results under different structural variants.

Moreover, we present the visualization results of different model variants in Figure 7 to provide a more intuitive comparison of their performance. As observed, line 1 shows the output of the baseline YOLOv8-Pose model, line 2 illustrates the results obtained after integrating the proposed ELA module, and line 3 displays the corresponding heatmaps generated by the YOLOv8-Pose-ELA model. It can be seen that, in certain local regions—such as the arms, elbows, and wrists—the prediction accuracy is significantly improved with the inclusion of the ELA module. This enhancement highlights the module’s ability to refine local feature representations and improve the keypoint localization accuracy, particularly in complex scenarios involving occlusions, motion blur, or low-resolution inputs. Overall, this visualization further validates the effectiveness and practicality of the ELA module in improving the robustness and precision of human pose estimation.

### 4.4. Ablation Analysis

To validate the effectiveness of our proposed method, we conduct a comprehensive comparative study involving several representative attention mechanisms, including CA [42], the convolutional block attention module (CBAM) [49], SE [43], and the inverted residual mobile block (iRMB) [50]. These modules are integrated into our framework individually to assess their influence on the overall performance. Furthermore, we systematically investigate the effects of inserting each module at different positions within the network architecture to understand the sensitivity of placement and its impact on the feature extraction capabilities.

In Table 3, columns 3, 5, 7, and 9 correspond to different positions within the backbone where various attention mechanisms—including CA [42], CBAM [49], iRMB [50], SE [43], and the proposed ELA—are inserted. Each configuration is designed to evaluate the impact of incorporating these modules at specific stages of the backbone network. The experimental setup described in Table 2 is based on the ELA-9 configuration, where the ELA module is placed at position 9 within the backbone. This architectural placement is also illustrated in the network diagram shown in Figure 6.

**Figure 8:**
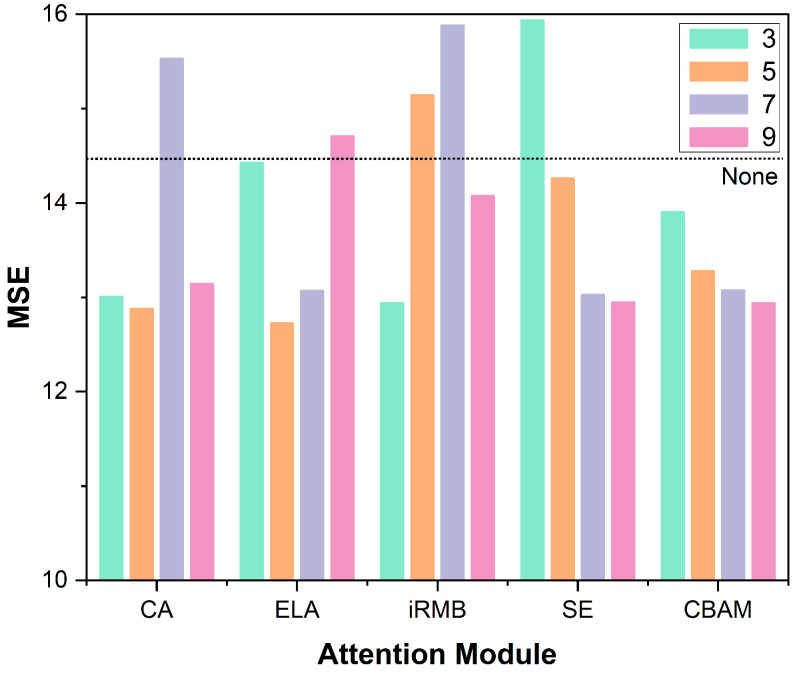
MSE comparison across attention mechanisms. Colors indicate module positions; the black dashed line marks the baseline without attention.

Figure 8 presents a bar chart that visualizes the quantitative results, comparing the performance of different attention mechanisms when integrated at various layers of the backbone network. As we can observe, CA [42] and ELA [22] achieve their best performance when inserted at the fifth layer, iRMB performs best at the third layer, and CBAM and SE yield optimal results when placed at the ninth layer. These trends suggest that the effectiveness of attention mechanisms is highly dependent on their integration depth within the network architecture. To better understand this phenomenon, we analyze the structural characteristics of each attention mechanism. CA [42], ELA [22], and iRMB incorporate spatial attention components that enable the model to more effectively capture and enhance positional information. These modules are particularly effective when applied in the early stages of the backbone, where spatial details and localization cues are more prominent and critical for accurate keypoint detection. In contrast, CBAM, despite combining both spatial and channel attention, places greater emphasis on channel recalibration and lacks the ability to model long-range spatial dependencies effectively. Consequently, it tends to perform better in the deeper layers of the backbone, where semantic abstraction and channel-based features dominate. Similarly, SE, a pure channel attention mechanism, adaptively learns the importance of each feature channel through global context modeling. This makes it more suitable for the later stages of the backbone, where feature representations are semantically richer and more abstract.

Based on these findings, we conclude that attention modules emphasizing spatial attention, such as ELA, CA, and iRMB, are more effective when integrated into the early stages of the feature extraction pipeline. At these stages, enhancing the model’s sensitivity to spatial and positional cues is crucial for accurate human pose estimation. In contrast, channel attention-based mechanisms like SE and CBAM are better suited for deeper layers of the network, where semantically rich features dominate. These mechanisms effectively refine feature representations by selectively emphasizing informative channels based on the global context.

Overall, in the context of human pose estimation, where the precise localization of keypoints is critical, spatial attention mechanisms, including ELA, iRMB, and CA, demonstrate a clear advantage due to their ability to strengthen fine-grained positional feature representations.

### 4.5. Comparative Experiments

In this section, we also conduct comparative experiments with several mainstream human pose estimation models, and the results are summarized in Table 4. Specifically, we employ three widely used evaluation metrics: the mean squared error (MSE), percentage of correct keypoints (PCK), and object keypoint similarity (OKS). These metrics collectively assess the accuracy of keypoint localization from multiple perspectives, i.e., the MSE captures the average pixel-level prediction error, PCK quantifies the proportion of correctly predicted keypoints within a predefined threshold, and OKS measures the alignment between predicted and ground truth keypoints while accounting for the object scale and keypoint visibility. This comprehensive evaluation framework enables a more objective and rigorous comparison across different models. As shown in Table 4, our proposed method achieves the lowest MSE and the highest PCK and OKS scores among all evaluated approaches. These results demonstrate the superior accuracy and robustness of our model in human pose estimation tasks, particularly under complex, domain-specific conditions such as those found in badminton scenarios.

**Figure 9:**
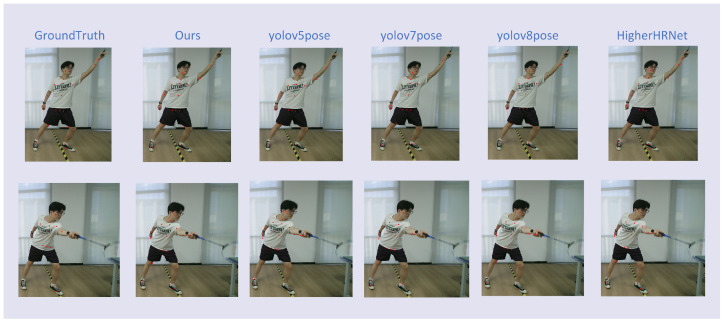
Effectiveness comparison of different networks. All input samples are originally at a resolution of 1920 × 1080, and the images are cropped accordingly.

Figure 9 further illustrates the predicted keypoints generated by different models for a series of badminton-specific actions. It can be observed that our method consistently produces predictions that are closer to the ground truth, particularly at critical limb joints such as the elbows and wrists. These joints are essential for accurate pose analysis in racket sports due to their high degrees of freedom and coordination demands. Moreover, we can observe that our model not only outperforms competing methods in quantitative metrics but also generates more anatomically plausible and visually consistent pose estimations in the qualitative comparison. These results validate the effectiveness of integrating the ELA mechanism into the YOLOv8-Pose framework. By enhancing the model’s ability to capture fine-grained spatial dependencies, especially in highly articulated regions, the ELA module plays a crucial role in improving the pose estimation accuracy under the complex and dynamic motion patterns characteristic of badminton gameplay.

### 4.6. Runtime Evaluation

In addition to accuracy, the runtime performance is a critical factor for human pose estimation models, especially in real-time sports analytics applications. To evaluate the efficiency of our proposed method, we conduct a comprehensive runtime analysis comparing our proposed model with several representative models, including HigherHRNet [37], LitePose-M [39], and some YOLOv8-Pose-based variants. All models are benchmarked using the same image input resolution of 640 × 640 pixels and are evaluated on a single RTX A6000 GPU and an Intel(R) Core(TM) i7-9700 CPU @ 3.00 GHz to provide GPU latency comparisons. The results are summarized in Table 5. As we can observe, our enhanced YOLOv8n-Pose model with ELA integration not only achieves an acceptable FPS but also maintains a competitive model size and computational complexity (GFLOPs). Despite the addition of the ELA module, which slightly increases the parameter count compared to other lightweight models, our approach still operates comfortably above the 25 FPS real-time threshold. These results confirm that our model offers a favorable trade-off between accuracy and efficiency, making it well suited for deployment in real-time, resource-constrained environments.

## 5. Conclusions and Discussion

With a specific focus on badminton scenarios, a customized human pose estimation dataset tailored to badminton, called xBHPE, is constructed. Extensive ablation studies on the xBHPE dataset demonstrate that integrating the ELA mechanism significantly improves the accuracy of keypoint localization. In addition, we analyze the impact of attention module placement within the network and identify general design patterns that contribute to optimal performance. We further highlight that the core components of our proposed framework, such as the YOLOv8-Pose backbone and the ELA mechanism, are model-agnostic and not restricted to badminton-specific scenarios. These modules can be readily adapted to other sports or human motion analysis tasks that involve dynamic and fine-grained body movements, such as tennis, volleyball, or basketball. Furthermore, the modular design of our attention integration strategy allows for straightforward customization to accommodate different skeletal structures, movement complexities, or sport-specific keypoints, with minimal changes to the overall network architecture. This flexibility makes our approach a promising candidate for broader applications in sports analytics and general human pose estimation tasks.

## Figures and Tables

**Table 1:** Kinectv2 camera parameters.

Hardware	Function
Color camera	Used to shoot color video images within the field of view
Infrared projector	Active projection of near-infrared spectrum, where the light reflected by the object can be read by the infrared camera
Depth (infrared) camera	Analyzes the infrared spectrum to create a depth image of the human body and objects within the visible range
Four-element linear microphone array	Built-in digital signal processing DSP and other components, while filtering background noise; can locate the direction of the sound source

**Table 2:** ELA performance comparison of different parameters.

Method	P	R	mAP50	mAP50-95	MSE
YOLOv8-Pose	1	1	0.995	0.991	14.4467
ELA-5-256-16	1	1	0.995	0.986	13.5158
ELA-5-256-32	1	1	0.995	0.990	15.5175
ELA-5-32-16	1	1	0.995	0.987	13.0794
ELA-5-32-32	1	1	0.995	0.990	13.3934
ELA-7-256-16	0.998	1	0.995	0.986	13.1269
ELA-7-256-32	1	1	0.995	0.989	13.3907
ELA-7-32-16	1	1	0.995	0.986	13.8766
ELA-7-32-32	1	1	0.995	0.988	13.3213

**Table 3:** Comparison of MSE values for CA, CBAM, and ELA modules inserted at different positions within the YOLOv8-Pose backbone.

Method	3	5	7	9
None	14.4467			
CA	13.0044	**12.8770**	15.5243	13.1416
CBAM	13.9023	13.2752	13.0737	**12.9372**
iRMB	**12.9374**	15.1394	15.8798	14.0726
SE	15.9351	14.2572	13.0243	**12.9437**
ELA	14.4220	**12.7215**	13.0665	14.7058

**Table 4:** Comparison of MSE, PCK, and OKS values across different networks on the xBHPE dataset.

Method	MSE	PCK@0.2	OKS
YOLOv5-Pose	14.6463	0.7286	0.8663
YOLOv7-Pose	14.0314	0.7211	0.8597
YOLOv8-Pose [40]	14.4467	0.7421	0.8713
YOLOv8-Pose-CA	12.8770	0.7348	0.8632
YOLOv8-Pose-CBAM	12.9372	0.7365	0.8664
YOLOv8-Pose-iRMB	12.9374	0.7371	0.8667
YOLOv8-Pose-SE	12.9437	0.7394	0.8701
HigherHRNet [37]	12.9670	0.7884	0.8796
LitePose [39]	13.8043	0.7568	0.8774
BlazePose [51]	13.1237	0.7602	0.8816
Ours	**12.7215**	**0.7793**	**0.8874**

**Table 5:** Runtime comparison of our method with other human pose estimation models.

Model	Params (M)	GFLOPs	FPS (GPU)	Real Time
HigherHRNet [37]	28.5	45.6	15.7	NO
LitePose-M [39]	4.5	4.8	63.1	YES
YOLOv8-Pose [40]	3.8	8.7	75.5	YES
YOLOv8-Pose-CBAM	4.1	10.1	68.9	YES
YOLOv8-Pose-iRMB	4.2	9.8	64.3	YES
**Ours**	**4.1**	**10.3**	**66.4**	**YES**

## Data Availability

Data will be available upon request to the corresponding author.

## Abbreviations

The following abbreviations are used in this manuscript:

ELA	Efficient local attention
AI	Artificial intelligence
AR	Augmented reality
VR	Virtual reality
HPE	Human pose estimation
RMPE	Regional multi-person pose estimation
CPN	Cascaded pyramid network
HRNet	High-resolution network
GCN	Graph convolutional network
PAF	Part affinity field
YOLO	You Only Look Once
BN	Batch normalization
CNN	Convolutional neural network
CA	Coordinate attention
SE	Squeeze-and-excitation
GN	Group normalization
SPPF	Spatial pyramid pooling fast
CBAM	Convolutional block attention module
iRMB	Inverted residual mobile block
MSE	Mean squared error
